# Enhanced Photoacoustic Gas Analyser Response Time and Impact on Accuracy at Fast Ventilation Rates during Multiple Breath Washout

**DOI:** 10.1371/journal.pone.0098487

**Published:** 2014-06-03

**Authors:** Alex Horsley, Kenneth Macleod, Ruchi Gupta, Nick Goddard, Nicholas Bell

**Affiliations:** 1 Institute of Inflammation and Repair, University of Manchester, Manchester, United Kingdom; 2 Manchester Adult Cystic Fibrosis Centre, University Hospital of South Manchester, Manchester, United Kingdom; 3 Department of Respiratory Medicine, Great Ormond Street Hospital, London, United Kingdom; 4 School of Chemical Engineering and Analytical Science, University of Manchester, Manchester, United Kingdom; 5 Department of Respiratory Medicine, Bristol Royal Infirmary, University Hospitals Bristol NHS Foundation Trust, Bristol, United Kingdom; University of Tübingen, Germany

## Abstract

**Background:**

The Innocor device contains a highly sensitive photoacoustic gas analyser that has been used to perform multiple breath washout (MBW) measurements using very low concentrations of the tracer gas SF_6_. Use in smaller subjects has been restricted by the requirement for a gas analyser response time of <100 ms, in order to ensure accurate estimation of lung volumes at rapid ventilation rates.

**Methods:**

A series of previously reported and novel enhancements were made to the gas analyser to produce a clinically practical system with a reduced response time. An enhanced lung model system, capable of delivering highly accurate ventilation rates and volumes, was used to assess in vitro accuracy of functional residual capacity (FRC) volume calculation and the effects of flow and gas signal alignment on this.

**Results:**

10–90% rise time was reduced from 154 to 88 ms. In an adult/child lung model, accuracy of volume calculation was −0.9 to 2.9% for all measurements, including those with ventilation rate of 30/min and FRC of 0.5 L; for the un-enhanced system, accuracy deteriorated at higher ventilation rates and smaller FRC. In a separate smaller lung model (ventilation rate 60/min, FRC 250 ml, tidal volume 100 ml), mean accuracy of FRC measurement for the enhanced system was minus 0.95% (range −3.8 to 2.0%). Error sensitivity to flow and gas signal alignment was increased by ventilation rate, smaller FRC and slower analyser response time.

**Conclusion:**

The Innocor analyser can be enhanced to reliably generate highly accurate FRC measurements down at volumes as low as those simulating infant lung settings. Signal alignment is a critical factor. With these enhancements, the Innocor analyser exceeds key technical component recommendations for MBW apparatus.

## Introduction

Multiple breath inert gas washout (MBW) is an old technique [1] that has been reinvigorated in the last decade by the recognition that this relatively simple test is a sensitive measure of lung physiology [2], [3]. MBW systems need to be able to measure tracer gas concentrations with a high level of accuracy at both the start and end of a washout, the end concentration conventionally being 1/40^th^ of that at the start. This requires a linear response profile of the analyser across a broad range, and a noise to signal ratio of at least 5% at end of washout [4]. In order to derive functional residual capacity (FRC), expired gas volumes are calculated by integrating the gas signal with flow. This process requires accurate and stable alignment of the two signals [5]. The other important feature of the gas analyser is that it must respond sufficiently quickly to be able to track a rapidly changing gas concentration signal [6]. Response time is typically quoted as T_90_, the time (in ms) between 10% and 90% of the stable gas signal response to a step change in gas concentration. Longer T_90_ response times lead to greater error in end tidal gas concentration and volume of gas expired, the magnitude of error being related to breathing rate [7]. Technological issues therefore become increasingly important and challenging as MBW is applied in younger children and infants, with much smaller tidal volumes and faster respiratory rates than adults.

A much needed boost to the standardisation of techniques has been provided by a recent expert consensus statement which addresses the key technological requirements for MBW devices (the gas analyser performance recommendations of which are summarised in Table 1) [4]. Although there are now a number of MBW devices available commercially, at present none of these is able to meet all the technical recommendations of the expert consensus. The majority of current commercial devices rely on nitrogen as the inert tracer gas, and oxygen as the washout gas, though these do not meet the recommendations for signal rise time and/or N_2_ signal measurement error [4], [8]. The vast majority of the study clinical data reported so far have involved the use of a mass spectrometer to measure 4% SF_6_ [9]–[11], and this device has been broadly described as the “gold standard” against which to compare other technologies [4], [12], [13]. Mass spectrometer-based systems, using the Amis 2000 (Innovision, Odense, Denmark) have been successfully used in all ages down to very young infants [14], and this is well-established technology in a number of research laboratories around the world [9]–[11]. From a practical point of view however these devices are bulky, expensive and temperamental. More constraining than this, the Amis 2000 is no longer manufactured, and indeed there are very few respiratory mass spectrometers still produced [15]. The third major technology used in clinical MBW testing is one based on the Innocor photoacoustic spectrophotometer (Innovision, Odense, Denmark) [16]. This has been used in adults and school age children [12], [17], [18], but concerns about the relatively long rise time of the gas signal have restricted its use in younger subjects [4], [19]. Until recently, this device has also required substantial in-house modification and separate analysis software to measure MBW outcomes such as lung clearance index (LCI).

**Table 1:** Summary of gas analyser performance consensus recommendations for MBW technology [4].

Component	Recommendation
Flow measurement	Volume accuracy ±3%
Gas analyser accuracy (linearity and signal to noise ratio)	Within 1% at start of washout and 5% at end
Gas analyser rise time (T_90_)	<100 ms
Data sampling frequency	≥100 Hz
Synchronisation of gas and flow signals	Accurate alignment within to 10 ms
Accuracy	FRC measurement accuracy ±5% of true FRC for ≥95% values

This paper describes modifications to the hardware of the Innocor device to improve the gas analyser T_90_, bringing this well within the recommendations and close to that of the mass spectrometer. We have then used a novel lung model, a refinement of a previously described system [8], [20], to assess accuracy of the Innocor device across a range of clinically relevant scenarios. Finally, we have investigated the effect of rise time and signal alignment on accuracy of FRC estimation. The primary aims of this study were to:

1. Improve the Innocor gas analyser response time to produce an MBW device that meets all of the consensus recommendations for MBW technology [4].
2. Define the accuracy of this system in terms of FRC estimation.
3. Define the impact of response time and misalignment of gas and flow signals on accuracy of FRC estimation.

A secondary aim was to explore the potential of the analyser to accurately measure FRC in settings of an infant lung model.

## Materials and Methods

The experiments were all performed on an open circuit apparatus. This is the same format that has previously been employed in clinical studies using the Innocor apparatus [16]–[18], [21], and is distinct from a recently described closed circuit prototype [22].

### Flow gas delay and response time

**Figure 1:**
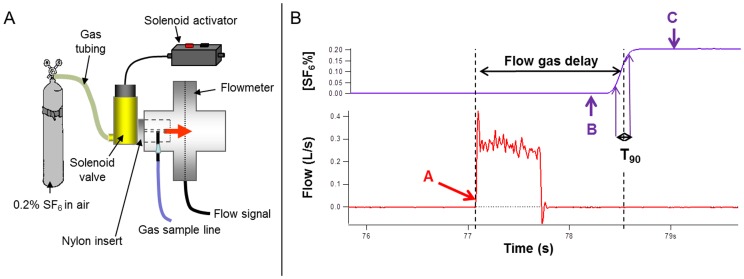
Measurement of flow gas delay (FGD) and response time. 1A: An instantaneous flow signal and square wave of SF_6_ was generated using an electronic solenoid-activated valve to direct a stream of 0.2% SF_6_ past the gas sample needle and onto the flowmeter mesh. 1B: Flow signal (red) showing a sudden rise when the solenoid is activated. Point A is the zero point for start of FGD measurement. SF_6_ signal is shown in purple with zero point (B) and SF_6_ plateau (C) identified. The software then identifies the 50% rise point, as the end of FGD, and the 10–90% rise time (T_90_).

The same method was used to measure flow gas delay (FGD) and T_90_ simultaneously. An electrically operated rapidly responding solenoid-activated valve (Clippard Inc, Ohio, USA) was connected to a supply of 0.2% SF_6_ in air (BOC special gases, Surrey, UK) at the inlet side, with gas flow of 4 Lmin^−1^. The outlet was connected to a custom-made nylon plug that fitted into the exhaust port of the flowmeter. Gas exiting the valve was directed over the gas sample needle and onto the mesh of the flowmeter. When the valve was activated, this produced an instantaneous spike in pressure, detected by the flowmeter, and a square wave change in SF_6_ from 0 to 0.2%. Custom built software, written using Igor Pro (Wavemetrics Inc., Oregon, USA), was used to identify FGD from the first up-spike in flowmeter pressure to the 50% maximum SF_6_ signal, minus time taken for the SF_6_ to traverse the 0.02 ml deadspace of the nylon plug, and with a fixed adjustment of −20 ms to allow for passage of gas through the valve and time to flow peak. T_90_ was defined as the time in ms for the SF_6_ signal to rise from 10% to 90% of plateau SF_6_ concentration. The apparatus and analysis software are illustrated in Figure 1. To separately assess the impact of oxygen on gas transit time, the same process was repeated using a mix of 1% SF_6_ in 94% oxygen and 5% N_2_O supplied from Innocor's own on-board gas supply (Innovision ApS, Odense, Denmark) at 8 L/min^−1^.

### Signal to noise

Signal to noise ratio (SNR) was defined as previously described as the ratio of the standard deviation of the gas concentration (the noise) to mean signal, expressed as a percentage [4]. SNR was assessed across a range of SF_6_ concentrations by serial dilution of a sealed gas sample. SNR was assessed over a minimum 20 seconds (2000 samples at 100 Hz) of a stable gas signal.

### Lung model

**Figure 2:**
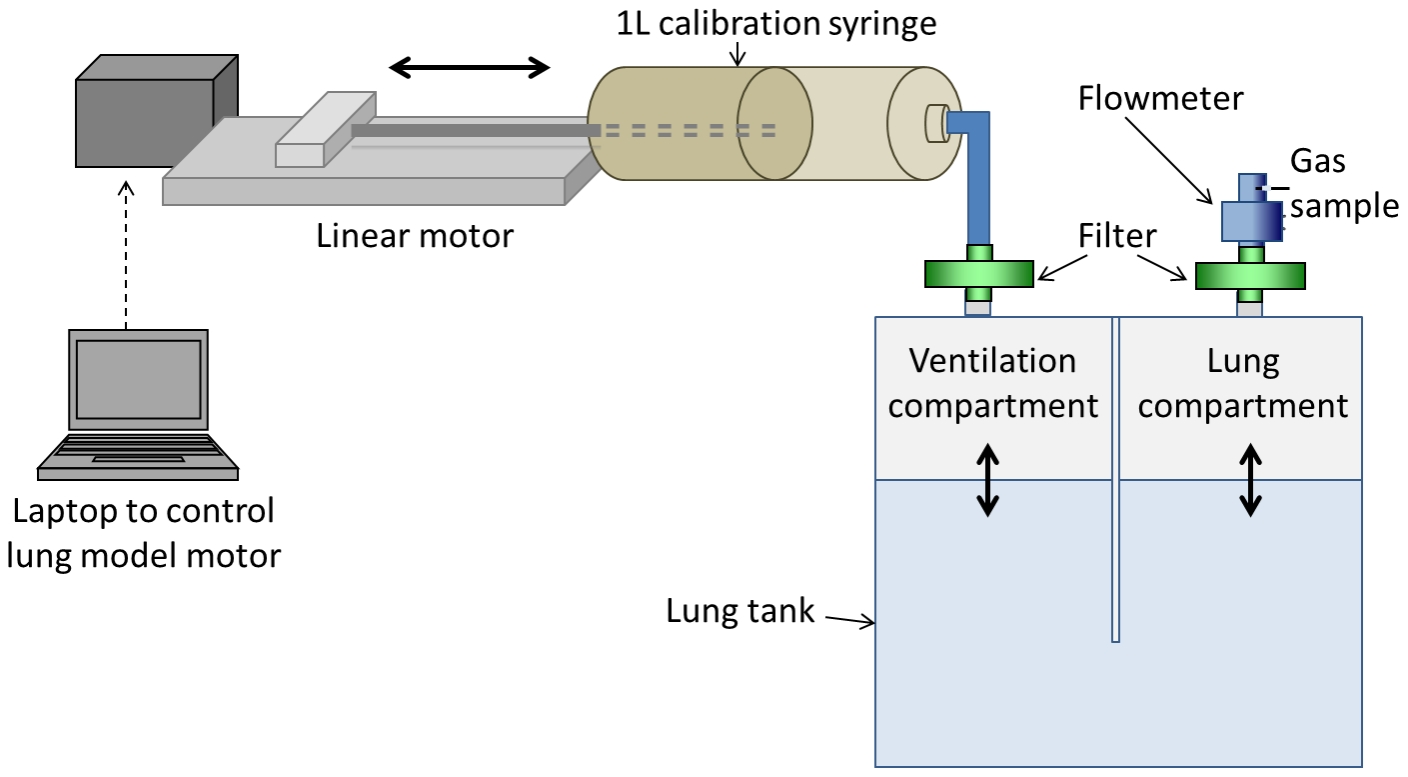
Diagram of lung model. A high-precision computer-controlled linear motor was used to drive a calibration syringe. This moved air into and out of the ventilation compartment of the lung tank. Water level in the lung compartment determined functional residual capacity (FRC) of the model whilst ventilation rate and volume were controlled by speed and excursion of the linear motor. A flowmeter and gas sample needle were connected to the lung compartment. During washin, a T-piece connected the flowmeter to the open circuit 0.2% SF_6_.

The lung model was based on one originally described by Brunner *et al.* and adapted for use in MBW device validation by Singer *et al.* [8], [23]. The model consisted of a sealed clear acrylic tank divided into two equal and communicating compartments, as illustrated in Figure 2. The internal dimensions of each compartment were 100×100×125 mm. The tank was filled with water to a pre-set level, according to the desired FRC. The “lung” compartment of the model was connected, via a bacterial filter, to the patient interface of the Innocor device. Pre-capillary deadspace (connector, filter, flowmeter) was calculated at 59 ml (combination of water fill volume and manufacturer-stated volumes, adjusted for connector overlap). The ventilation compartment of the model was connected via a filter to a 1 L calibration syringe (Hans Rudolph Inc, Kansas, USA) attached to a linear actuator (Bosch Rexroth AG, Schweinfurt, Germany). The position, speed and acceleration of the linear motor were controlled by a laptop computer running the manufacturer's own software. Repeatability of the linear motor is 0.2 mm (manufacturer's own data), which represents 0.06 ml of the 1 L syringe volume. Further data on lung model precision are given in the supporting information file S1. A distinct advantage of this over the previously described lung model, which drove the ventilator compartment by changing the pressure settings on a clinical ventilator [8], [20], is that this allows very precise adjustment of the speed and waveform of the breathing pattern as well as precise identification and accurate replication of the end expiratory water level. Accuracy of water level identification was estimated to be within 0.5 mm, reading from a ruler affixed to the internal wall of the lung compartment, or 6.25 ml. In this *in vitro* system, the model was not heated. Temperature and humidity of both expired air and that within the lung chamber were compared using a digital thermohygrometer (ATP Instrumentation Ltd, Leicestershire, UK), and were found to be the same, so no additional volume correction was applied.

### Infant Lung Model

A modification to the model described above was used to test the performance of the Innocor gas analyser against infant lung settings. A second smaller lung tank was used, along with a shorter connector and smaller filter (9070/01, Air Safety Limited, Morecambe, UK). The same flowmeter was used, but with the flow output re-linearised, using the on-board software, to take into account the new filter. Total apparatus and connector pre-capillary deadspace of this system was 48 ml; post capillary deadspace was unaltered at 2.5 ml. FRC was set at 250 ml, respiratory rate 60/min and tidal volume 100 ml in order to simulate recognised infant breathing parameters [24]. The deadspace of this apparatus was not optimised for infants (and instead reflects the available flowmeter, connectors and filters), but the purpose of this experiment was to investigate whether the improved Innocor system could perform accurately at these settings, and this was not intended as the definitive system for use in infants.

### Washout testing

Washin was performed from an open circuit consisting of two limbs of 1.5 m long ventilator tubing connected to the flowmeter via a T-piece (both from Intersurgical Ltd, Berkshire, UK). One limb was connected to a supply of 0.2% SF_6_ in air, in line with a reservoir bag, and the other was vented to exhaust. During washin, gas supply was provided at a sufficient rate to prevent inspiration of room air. Once fully washed in (inspired and expired [SF_6_] differ by <1%), the T-piece was rapidly removed during expiration. Expired tracer was dispersed by use of a fan. Washout was discontinued, and FRC calculated, from the point where end tidal SF_6_ fell to <0.005% (i.e. 1/40^th^ of the starting concentration), as described in the consensus statement [4].

Custom built software for offline washout analysis was written using Igor Pro (Wavemetrics Inc., Oregon, USA). This applied similar analysis protocols to those used in earlier studies [16], [18], with the following improvements: 1) adjustment was made for ambient SF_6_; 2) reinspired SF_6_ was measured and taken into consideration by integrating inspiratory flow with the aligned SF_6_ signal. Analysis was in line with software recommendations [4].

### Modifications to Innocor

The open circuit washout system was fundamentally the same as that described in the original clinical paper [16]. A low volume unheated flowmeter (Model 4179, Hans Rudolph Inc, Kansas, USA) was connected to a bacterial and viral filter (Model 4222/701, Air Safety Ltd, Morecambe, UK). The gas sample line was sited on the distal side of the flowmeter, with a post capillary deadspace of 2.5 ml. Data on resistance are presented in the supporting information file S1. Gas sample flow was measured using a digital flowmeter (TSI Instruments Ltd, Buckinghamshire, UK). Gas and flow data sampling frequency of the Innocor device was 100 Hz and a daily calibration was performed to confirm that flowmeter accuracy was within 3% (typically within ±1%).

Gas analyser response time (T_90_) is affected by three main components:

1) The intrinsic response time of the photoacoustic gas analyser (PGA) itself. This was assessed by connecting the analyser directly to the solenoid.

2) Gas signal dispersal in the Nafion gas sample line and connectors. This was assessed by optimising the connectors and by sequentially shortening the 170 cm gas sample line in 20 cm steps, taking care to ensure a good seal of the cut ends. Previous studies have confirmed that the minimum length for equilibration of humidity and temperature with room air was 40 cm [25], though this length is too short for practical use.

3) Gas signal dispersal in the Oxigraf oxygen analyser, placed in series with the PGA. This was assessed by re-routing the gas sample line to connect directly to the PGA, which also removes the ability of the system to measure O_2_.

## Results

### Response time improvement

**Figure 3:**
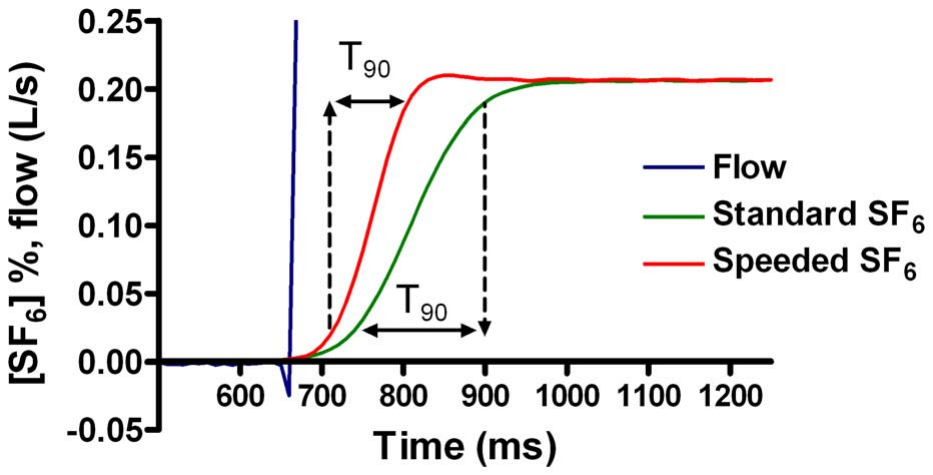
Impact of hardware improvements on SF_6_ response time (T_90_). Aligned flow and SF_6_ signals are shown for a standard Innocor (T_90_ = 154 ms), and an improved version, labelled “Speeded SF_6_” (T_90_ = 88 ms). Double headed arrows indicate the time between 10% and 90% plateau SF_6_ response.

The impact of the response time improvement steps is summarised in Table 2 and illustrated in Figure 3. The intrinsic response time of the PGA was 56 ms. Almost 30 ms of the total 155 ms was caused by the Oxigraf analyser. Shortening the Nafion gas sample line produced small reductions in T_90_, but had a more significant impact on FGD. In contrast, introduction of an optimised connector design reduced the response time by 25 ms, with relatively trivial impact on FGD. In the final system, the effect of reducing Nafion to 90 cm, removing the Oxigraf, and optimising the connections was a T_90_ of 88 ms. With a definitive seal to the Nafion gas sample line, this was reduced further to 85 ms. This is illustrated in Figure 3. Gas sample flow of the modified system was measured at 137 ml/min.

**Table 2:** Improvements to Innocor gas analyser rise time.

	Condition		Mean T_90_ (ms)	SD T_90_ (ms)	N repeats	Mean (SD) FGD (ms)
1	Standard system (170 cm gas sample line)		154.6	0.95	41	1376 (3.7)
2	Oxigraf removed		125.5	1.2	19	1026 (2.3)
3	Nafion gas sample line length	150 cm	121.1	1.96	25	932 (2.6)
		130 cm	119.0	2.12	23	860 (3.7)
		105 cm	115.9	2.36	44	782 (3.3)
		90 cm	113.6	2.11	20	708 (2.2)
4	Custom-optimised connectors		88.6	1.6	28	671 (3.6)
5	Photoacoustic gas analyser only		55.9	0.65	8	-

Effect of modifications to Innocor hardware on rise time (T_90_) and flow gas delay of SF_6_ signal. Steps were performed sequentially in the numbered order. FGD - flow gas delay.

There was no difference in the measured SF_6_% between humidified and dry samples with either full length (170 cm) Nafion sample line, or the shortened sample line (87 cm), see Table S1 in supporting information file S1.

### Flow gas delay

Accurate and reproducible flow and gas signal alignment is vital for accurate integration of expired gas volumes, and it is recommended that error in this measurement be <10 ms [4]. Reproducibility of FGD was assessed over n = 140 FGD measurements, performed at 3 different time points on the same day using the solenoid-activated valve. Overall FGD was 657 ms, standard deviation was 3.9 ms, maximum 666.7 ms and minimum 646.4 ms. The improvement over the data in Table 2 is due to the improved and permanent seal used to join the cut ends of the Nafion gas sample line. At the three time-points spread over 7 hrs, mean (SD) FGD was 659.1 (2.9), 656.2 (2.8) and 653.8 (3.7) ms. Environmental conditions in the test room were stable, and the falling FGD likely relates to warming of the immediate environment/gas sample line.

On a separate occasion, FGD measurements were repeated to assess the impact of oxygen on the delay time. FGD measurements were first performed as described above using 0.2% SF_6_ in air as the test gas. Measurements were then repeated using 1% SF_6_ in 94% O_2_ and 5% N_2_O (supplied from the on-board test gas cylinder), with the flowmeter attached to a flowpast circuit containing 100% O_2_ at 3 Lmin^−1^. Mean (SD) FGD was 651.4 (2.5) ms (n = 26) for SF_6_ in air, and 716.1 (3.2) ms (n = 25) for SF_6_ in O_2_, (p<0.00001)

### Signal to noise ratio

**Figure 4:**
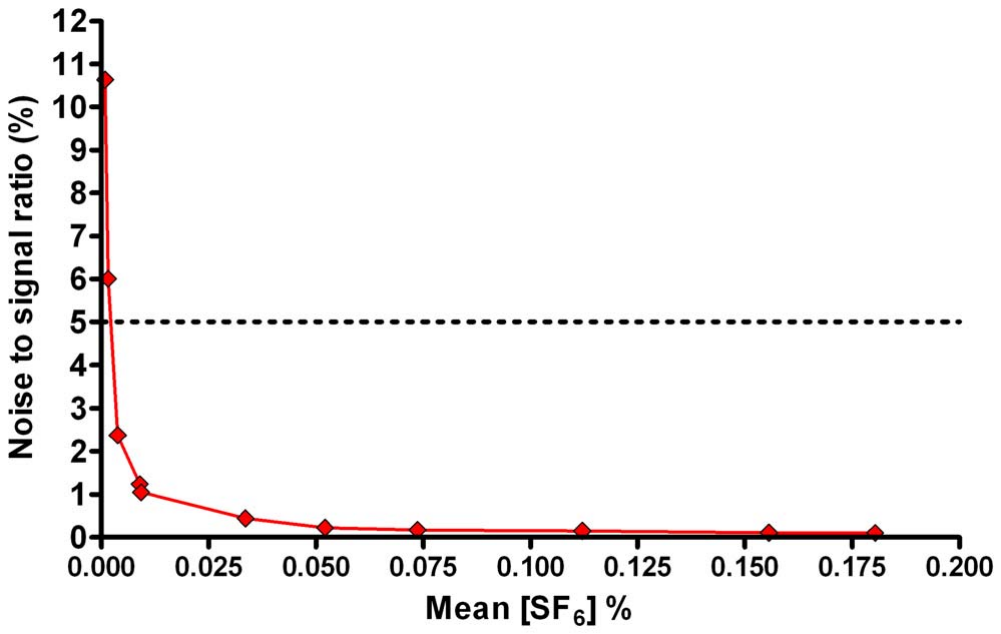
Signal to noise profile of the Innocor gas analyser. Mean SF_6_ concentration versus signal to noise ratio (defined as standard deviation of the signal as a percentage of mean signal for a minimum of 2000 samples). Horizontal dotted line represents the 5% signal to noise ratio deemed acceptable from a technical point of view.

SNR varied from 0.1% at a mean [SF_6_] of 0.18%, to 10.6% at a mean [SF_6_] of 0.0009%. Non-linear regression of the graph of SNR vs SF_6_ concentration (Figure 4) generated the equation SNR = 0.0234.*x* ^−0.842^ (where *x* is [SF_6_]) with an R^2^ of 0.99. According to this, SNR is below 5% (the level deemed technologically acceptable) for concentrations of SF_6_ above 0.002% (see Figure 4).

### Accuracy of Innocor system

**Figure 5:**
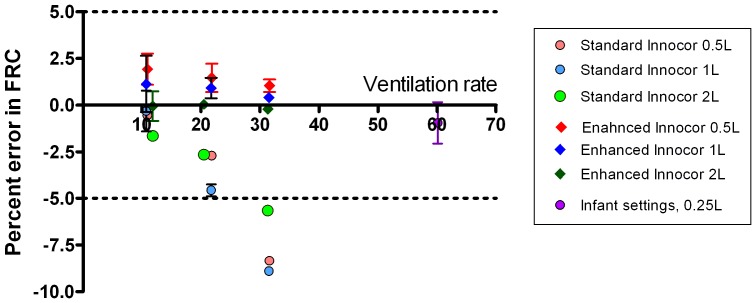
Accuracy of Innocor gas analyser at calculating lung volumes at different ventilation rates and volumes. Effect of increasing lung model ventilation rate on accuracy of FRC calculation from multiple breath washout for different FRC's. Effect of analyser performance is shown by comparing the speeded system (T_90_ 88 ms) to that of the standard system (T_90_ 154 ms). Data are shown as mean and 95% confidence interval, horizontal lines denote the 5% limits of acceptable error in FRC determination [4]. The “infant” settings at 60minute^−1^ refer to the smaller lung model, run on the speeded system only.

A minimum of 5 washouts were performed at each of three lung model ventilation rates (10–32 min^−1^) and three FRCs (0.5, 1 and 2 L). These were performed using the ‘speeded’ system (with a T_90_ of 88 ms) and repeated using the same system but with the T_90_ increased by addition of an extra filter (Camlab, Cambridge, UK) to the gas sample line in order to slow the rise time to 154 ms (i.e. the same as the standard Innocor setup). Since FGD was also increased by addition of the extra filter, the system-specific FGD was used for each analysis. Results are presented in Table 3. FRC accuracy is quoted as percentage of the lung tank volume, excluding the apparatus deadspace. Accuracy was good at slow ventilation rates across all three FRC volumes and in both the fast and slow T_90_ systems. 100% of all repeats at this rate produced an error of less than 5%. This accuracy was maintained at faster rates for the fast T_90_ system, where 100% of all washouts at all speeds (n = 49) generated an error in FRC of under 5% (range −0.9 to 2.9% error). For the slow T_90_ system however, error was greater at ventilation rate>20 min^−1^, and all washouts at rates >30 min^−1^ showed error in FRC of >5%. These data are also presented in Figure 5.

**Table 3:** Accuracy of standard and enhanced Innocor gas analyser.

System T_90_ (ms)	FRC (L)	Mean (SD) percentage error in FRC		
		Slow ventilation (10–12 min^−1^)	Medium (20–22 min^−1^)	Fast (31–32 min^−1^)
**Fast 88**	0.521	1.87 (0.77)	1.48 (0.73)	0.79 (0.27)
	1.011	1.15 (1.2)	0.94 (0.46)	0.40 (0.18)
	2.001	−0.06 (0.63)	0.04 (0.15)	0.21 (0.95)
**Slow 154**	0.521	0.07 (0.30)	−2.72 (0.81)	−8.33 (0.36)
	1.011	−0.30 (0.87)	−4.58 (0.24)	−8.89 (0.21)
	2.001	−1.65 (0.87)	−2.67 (0.12)	−5.91 (0.34)

Summary of error in functional residual capacity (FRC) calculated by multiple breath washout for a lung model at different FRC and ventilation rates. FRC are shown excluding the equipment dead space of 59 ml, and error is quoted as a percentage of the lung model FRC only (excluding deadspace). Precise ventilation rates varied with the lung model settings specific to each FRC and were 10.9, 21.8, 31.6 min^−1^ for 0.5 L FRC, 10.8, 21.7, 31.5 min^−1^ for 1 L FRC, and 11.8, 20.5, 31.3 min^−1^ for 2 L FRC. T_90_: 10–90% rise time of the gas analyser.

### Effect of rise time and signal alignment

**Figure 6:**
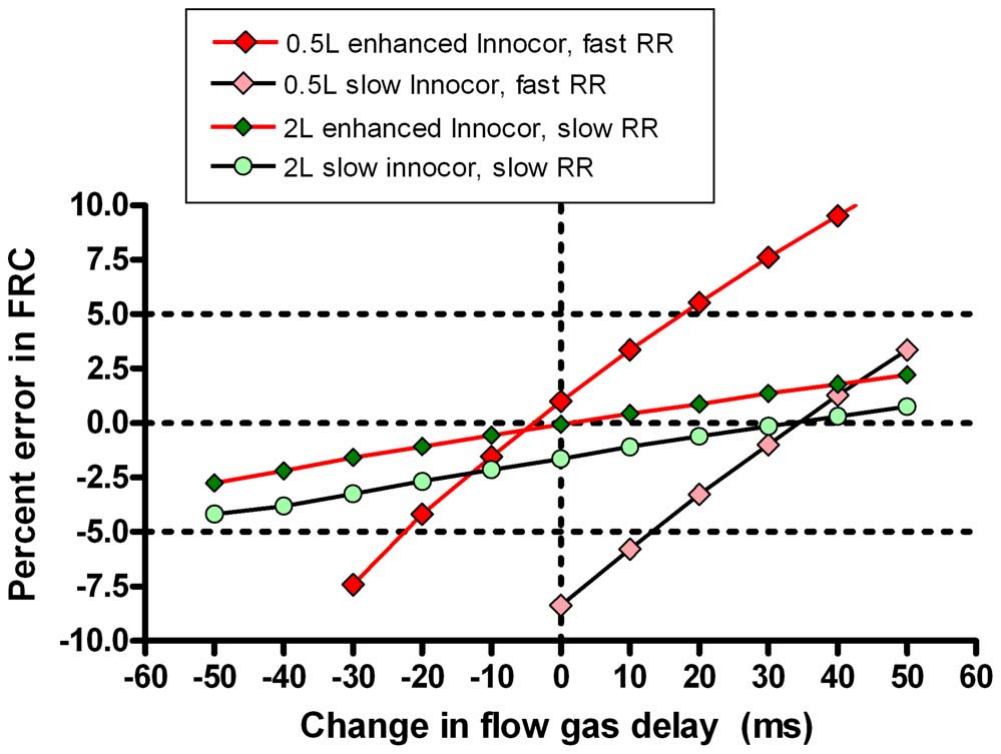
Effect of flow and gas signal misalignment on accuracy of lung volume calculation. Two lung model multiple breath washout scenarios are presented: small functional residual capacity (FRC) and rapid ventilation (red diamonds) and large FRC slow ventilation (green circles). Washouts were performed on a speeded system (red joining lines) or a standard system (black joining lines). Data points are means of at least 5 repeats. Horizontal dotted lines represent the 5% limits of acceptability for FRC determination; vertical dotted line represents the correct signal alignment. Slope of the graph is a measure of error sensitivity. FGD  =  Flow gas delay.

**Figure 7:**
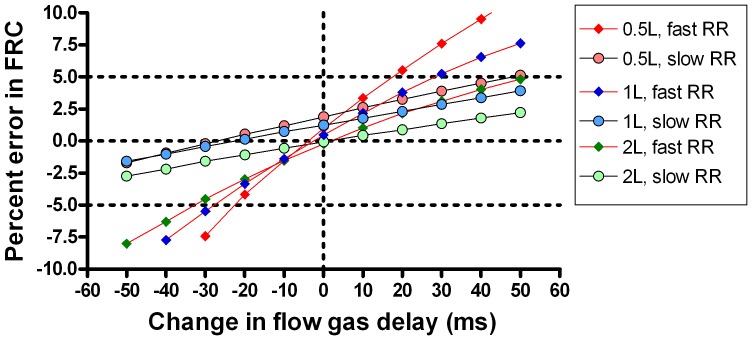
Importance of accurate flow-gas signal alignment in different lung model scenarios. The effect of increasing ventilation rate (red joining lines) and signal alignment on accuracy of a lung model, generated using the speeded Innocor analyser. Slope of error versus signal misalignment was increased by smaller lung volumes and faster ventilation rates. Horizontal dotted lines represent the 5% limits of acceptability for functional residual capacity (FRC) determination; vertical dotted line represents the correct signal alignment. RR: respiratory rate, FGD: flow gas delay.

**Figure 8:**
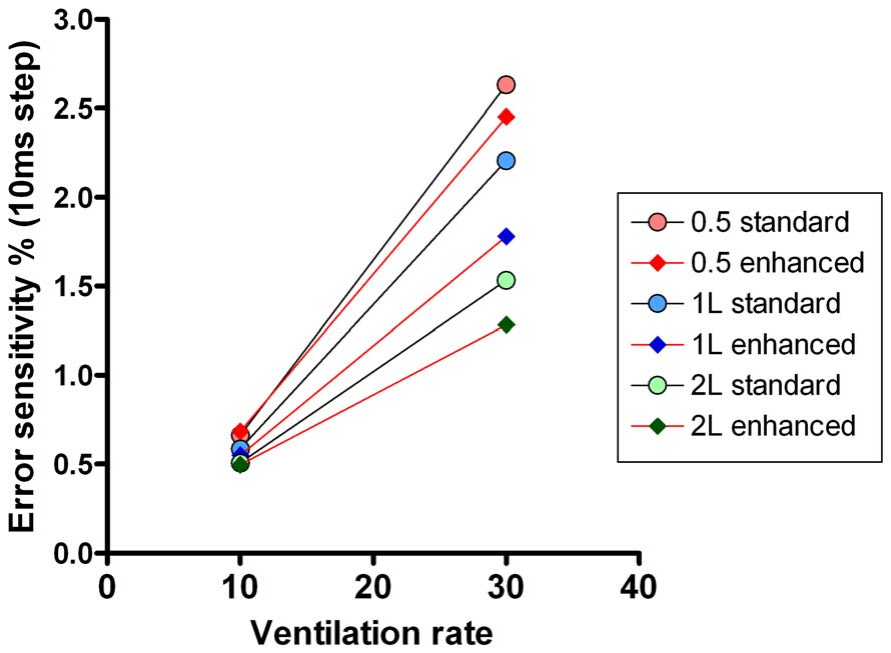
Error sensitivity of lung volume calculation. Error sensitivity of the two different Innocor systems (speeded with T_90_ = 88 ms, and slow with T_90_ = 154 ms) at two different lung model ventilation rates. Performances of the speeded system are joined by red lines, those of the slow system by black lines. Error sensitivity was defined as the % error in FRC that would be caused by a 10 ms (single sample step) mis-alignment in flow gas delay.

The washouts presented in Table 3 were also analysed with adjustments to the FGD in order to explore the impact under different conditions of flow and gas signal mis-alignment on washout accuracy. Each washout was analysed at 10 additional FGD alignments in 10 ms steps from −50 ms to +50 ms of the measured FGD. The most challenging scenario from a technical point of view is that of the small FRC (0.5 L) and fast ventilation rate (30 min^−1^), whereas the least technically challenging scenario is the 2 L FRC ventilated at 10 min^−1^. A comparison of the effect of FGD misalignment on these two scenarios, for both the speeded (T_90_ = 88 ms) and slow (T_90_ = 154 ms) systems is presented in Figure 6. The slope of the graphs in Figure 6 represent the error sensitivity of the system, i.e. the degree to which errors in signal alignment affect accuracy of FRC determination [5]. This is much steeper when the model is ventilated at a fast rate. Figure 6 also illustrates how slower response time can also be compensated for by shifting the FGD of the standard system by around 30–40 ms. Figure 7 illustrates the performance of the speeded system at different FRC and fast and slow ventilation rates. Although the absolute error remains small at faster ventilation rates, the slope of the error-FGD misalignment curve increases with increasing rate. This error sensitivity is summarised in Figure 8 at fast and slow ventilation rates for both the speeded and slow analyser systems. Error sensitivity was low for both systems at low ventilation rates, but increased with smaller FRC and faster ventilation, and was greater in the “slow” system with longer T_90_.

### Performance against infant lung settings

Ten washouts were performed with an FRC of 250 ml, mean (SD) tidal volume 101 (0.002) ml, at a rate of 60.1 minute^−1^ using the performance-enhanced Innocor. Analysis settings were as for the previous lung model washouts. Mean (SD) measured FRC (after subtracting deadspace) was 247 (0.005) ml. Mean (SD) error in FRC was -0.95 (1.6)%. All measurements were within the 5% error range required by guidelines; range of error was −3.8 to 2.0%. These data are shown in Figure 5 alongside the other lung model washouts.

## Discussion

In this study we have shown that not only can the response time of the Innocor analyser be reduced to well under the 100 ms target required by the MBW technical guidelines [4], but that this results in a system with enhanced accuracy at a range of clinically relevant lung model settings. The response time described here is 10% faster than the previous best reported for Innocor [25], but importantly this has been achieved with a long enough gas sample line to allow the system to be practically applied in multiple breath washout measurements. This new improvement is largely the result of custom-modified connectors that reduce gas front dispersal in the gas sample system. The resulting response time is now close to that of the Amis 2000 mass spectrometer (64 ms) [16] conventionally accepted as the optimal analyser for MBW tests [4], a device that is no longer available. Response time is also similar to or better than the majority of other respiratory mass spectrometers [15].

In addition to improving the performance of the analyser, we have used a precision lung model to demonstrate that this produces highly accurate measurements of FRC at low lung volumes and fast respiratory rates, down to values representative of those encountered in infants and preschool children [24]. Accuracy of FRC determination is important not just in its own right, but also in deriving accurate measures of LCI that are not affected by technical limitations at smaller lung volumes [20].

The selection of 100 ms as the cut off for system response time (T_90_) is to a certain extent arbitrary, though has now been enshrined in at least two sets of international expert consensus guidelines [4], [26]. Our data illustrate the importance of a short response time in rapidly-ventilated systems, though are not able to support the selection of any specific response time. At faster rates it is likely that the differences in FRC error and error sensitivity will continue to increase.

The lung model data reinforce the importance of accurate flow and gas signal alignment on lung volume calculations, which is not specific to this apparatus. At slow ventilation rates, as seen in adults, FRC calculation is relatively insensitive to even fairly substantial misalignment of the gas and flow signals (up to +/−50 ms, see Figure 7). At faster rates however, the relationship between error and signal misalignment is very much steeper. This principle has been recognised for some time [27], but has not previously been demonstrated in a fast responding system. The recent consensus statement recommended that FGD be accurately measured within 10 ms, or 1 sample point, though also acknowledged a lack of published evidence for this specific recommendation [4]. These data support the need for this level of accuracy in FGD determination, particularly in systems intended to be used at ventilation rates above or around 30 per minute. The highly reproducible FGD generated by the electrically-activated solenoid valve performs well within this level of repeatability, though the data also support the practice of repeat FGD assessment if testing takes place over several hours.

An important aspect of FGD is that this will change with the viscosity of the sampled gas. The Innocor system, by using only trace SF_6_, is subject to only very small changes in viscosity due to differences in inspired O_2_, CO_2_, humidity and temperature. For systems using 100% O_2_ as the washout gas this is potentially much greater, and dynamic gas signal delay adjustment is required to prevent potentially significant flow and gas signal misalignment of up to 55 ms. This phenomenon has already been described for the mass spectrometer [23], but also applies to any device involving a sidestream analyser and large changes in gas composition.

An interesting additional observation on gas signal alignment is shown in Figure 6. This demonstrates that not only is the sensitivity of FRC measurements to flow and gas signal alignment low at adult ventilation rates and FRC, but also that accuracy at faster rates is very similar to the fast-responding system if an adjustment is applied to the signal alignment. This is the same approach that has been used previously [16]–[18]. The advantage of the fast-responding system however is that it is also able to integrate re-inspired tracer gas volumes accurately, and therefore retains high levels of accuracy if additional post capillary deadspace is present. This is important for smaller subjects where the reinspired volumes cannot be so easily ignored as in adults.

The calculation of FRC to within 5% of true value is accepted as an important measure of system accuracy [4], [24]. The dependence on highly accurate flow and gas signal alignment at faster rates and with smaller volumes may explain why Gonem *et al.* found a broader range of accuracy for FRC estimation using an Innocor analyser (95% limits of agreement −4.6% to 3.9%), and also observed that accuracy deteriorated below FRC of 1500 ml [20]. In this study, the authors removed the Oxigraf analyser to enhance response time, which based on our own data is likely to be around 120 ms. FGD calculation was also different from ours and relied upon reinspired tracer, which is dependent on inspiratory flow and assumes that gas signal fall time is the same as rise time. The errors in the accuracy data they presented are reminiscent of imperfect gas and flow alignment, since deterioration was also seen at larger FRC. Although they did not report a dependence of error on ventilation rate, the range tested was only from 12 to 24 breaths per minute, and may have been too small to detect this. Different offline analysis software was used, but both systems apply conventional algorithms to integrate the flow and gas signals, both in expiration and inspiration.

Detailed *in vitro* accuracy data across the range described here are also available for the Exhalyzer multiple breath nitrogen washout (MBNW) device (Eco Medics AG, Duernten, Switzerland) [8]. For FRC of over 500 ml, the error in FRC calculation for this MBNW system was for the most part less than 5% [8], but the range was much broader than that reported here (95% confidence intervals from +4.7% to −4.0%, compared to all measurements within 0.9 to −2.9% for the Innocor analyser). For smaller FRC volumes however, accuracy in the MBNW study deteriorated. These MBNW data included some washouts with an FRC down to 100 ml, but even allowing for this the Innocor analyser was substantially better at accurately measuring lung volumes of 250 ml, with all measurements within 4% of the true FRC and an error range less than half that reported for MBNW. More recently, similar data have also been published using an ultrasonic molar mass system to measure either 4% SF_6_ in a mainstream apparatus or 20% Helium via a sidestream [28]. As with the MBNW apparatus, accuracy fell at lower FRC and was outwith the +/−5% limits for FRC less than 600 ml using the sidestream system.

All three of these prior studies have used the same lung model to validate their MBW apparatus [8], [20], [28]. A difference between these and the current study is that the lung model used here was run at room temperature. Since the intent of this study was accuracy of *in vitro* FRC determination, using a range of respiratory rates and volumes, external heating of the lung model to mimic BTPS conditions was deliberately not incorporated. The decision not to use heating may be seen as a weakness, since this is an aspect of lung models recommended by the consensus statement on LCI technology [4]. However it became apparent during initial testing that it was not possible at rapid ventilation rates to guarantee a stable temperature and humidity profile in the expirate, and nor do conventional thermohygrometers provide a sufficiently fast response to allow dynamic adjustment. This is not unexpected: the use of heating introduces potential variability of BTPS correction based upon gas volumes, gas mixing, and ventilation rate that are more relevant in a single chamber lung model than in the human lung. In this regard the recent consensus recommendation that all systems be tested under BPTS conditions may not be helpful. For ultrasound based systems, where temperature and humidity influence the measurement of flow, this is perhaps more relevant. In the current case however, where the gas sample is delivered to the analyser under ambient conditions, BTPS correction is only relevant in terms of adjusting for discrepancies in volume measurement due to temperature falls between lung chamber and flowmeter. The use of a heated model, and the inconsistencies that this introduces, may have contributed to the reduction in FRC accuracy seen in other studies at faster ventilation rates [8], [20], [28]. For a final commercial apparatus, incorporating commercial analysis software, the recommendation that the model be heated remains valid, though care is needed to ensure that this potential limitation of lung model heating is taken into account. We consider the current study to be a fair assessment of the performance of the photoacoustic gas analyser, and complimentary to the work by Gonem *et al.* demonstrating accuracy of FRC estimation using an Innocor analyser with a heated lung model at slower ventilation rates [20].

A further difference is that these earlier studies used a clinical ventilator to drive pressure changes in the lung model. Particularly at higher rates and smaller volumes, it is unlikely that this will be as accurate and reproducible as the precision linear motor described here, and may also have contributed to the loss of accuracy at these settings.

It should be emphasised that the system described here is not intended as a definitive infant MBW apparatus. The deadspace has not been optimised for this, and reflects the limitations of the available filters and connectors. Instead we have sought to demonstrate that the Innocor analyser is accurate at fast ventilation rates, and small lung volumes, throughout and beyond those likely to be encountered in young children and down to settings representative of older infants.

A potential disadvantage of the enhanced system described here is that it is no longer able to measure expired O_2_. This is however of limited relevance for the intended use of MBW testing in those with relatively well preserved lung function. Gas sample flow was increased modestly by the modifications to 137 mlmin^−1^, which compares to 20 mlmin^−1^ for the mass spectrometer-based systems and 200 mlmin^−1^ for the Exhalyzer MBNW apparatus [4]. As before, the potential of this to interfere with washout volumes has been dealt with by placing the gas sample line distal to the flowmeter. At present, this system is in a research configuration, and has not been tested against Innocor's own LCI analysis software. Finally, until further assessments have been completed, the hardware alterations mean that previous normal range data may no longer be accurate, and these assessments need repeated.

In summary we have described the enhancement of the Innocor gas analyser to produce a system capable of meeting the key technical component requirements for MBW apparatus. We have extended earlier observations [20] to demonstrate the accuracy of the system across a range of clinically relevant lung model settings, down to those simulating larger-infant parameters, and have shown it to have excellent accuracy within 4% of the target FRC. This has involved the use of an improved lung model that can also be programmed to deliver highly accurate small tidal volumes and fast respiratory rates. The Innocor analyser thus offers a genuine alternative to the mass spectrometer for many applications. Future applications in younger subjects will benefit from these performance enhancements, and avoid the need for 100% oxygen as the washout gas.

## Supporting Information

File S1 **Online supplement of supporting data.**
(DOCX)
