# Supplementary material for: Enhanced Photoacoustic Gas Analyser Response Time and Impact on Accuracy at Fast Ventilation Rates during Multiple Breath Washout
Source: PLoS One. 2014 Jun 3;9(6):e98487. doi: 10.1371/journal.pone.0098487 (PMC4043749; doi:10.1371/journal.pone.0098487)
Supplement: File S1 — Online supplement of supporting data. (DOCX) [file pone.0098487.s001.docx]

**Enhanced photoacoustic gas analyser response time and impact on accuracy at fast ventilation rates during multiple breath washout.**

Alex Horsley^1,2^, Kenneth Macleod^3^, Ruchi Gupta^4^, Nick Goddard^4^, Nicholas Bell^5^

1: Institute of Inflammation and Repair, University of Manchester, Manchester, UK

2: Manchester Adult Cystic Fibrosis Centre, University Hospital of South Manchester, Manchester, UK

3: Department of Respiratory Medicine, Great Ormond Street Hospital, London, UK

4: School of Chemical Engineering and Analytical Science, University of Manchester, Manchester, UK

5: Department of Respiratory Medicine, Bristol Royal Infirmary, University Hospitals Bristol NHS Foundation Trust, Bristol, UK

**File S1: Online supplement of supporting information**

*Resistance*

Resistance was measured using a Druck digital pressure transducer, model DPI 705 (GE Measurement and Control, Atlanta, USA). Pure air was applied, and flow increased stepwise to 750ml/s for the adult filter and 500ml/s for the paediatric filter. The system was configured in an open circuit, with bacterial filters in place and the T-piece removed. Resistance was predominantly determined by the filter. When using the adult filter, maximum resistance across the range was 0.33kPa/L.s at 750ml/s of air flow, whilst for the paediatric filter maximum resistance across the range studied was 0.39kPa/L.s at 500ml/s air flow.

*Repeatability of the lung model*

The linear actuator provides high precision in the placing of the syringe plunger. The manufacturers quote the precision of the motor to be 0.2mm. In other words the motor will place the plunger within 0.2mm of the set point each time. This represents an accuracy of 0.06ml of the 1L syringe volume. It has not been possible to independently confirm the manufacturer’s figures for this as the mechanical precision is greater than the accuracy of the flowmeter (+/- 1%). Instead, measured tidal volumes (Vt) were retrospectively examined during the lung model washouts at the fastest settings (31-32 breaths per minute). Individual breath volumes were rounded to the nearest 1ml by the analysis software, so precision greater than this has not been possible.

For the smallest adult lung model settings (FRC 500ml), Vt varied between 196 and 197ml across the 5 washouts. Mean Vt was 196.7ml, SD 0.48ml, CoV 0.24%.

For the medium adult lung model (FRC 1L), Vt varied between 293 and 294ml. Mean Vt was 293.2ml, SD 0.36ml, CoV 0.12%.

For the largest settings (FRC 2L), Vt varied between 486 and 487ml. Mean Vt was 486.2ml, SD 0.36ml, CoV 0.07%.

The rounding of breath volumes to the nearest 1ml means that although the range of measured Vt is only 1ml the repeatability (in terms of Vt CoV) appears poorer at small volumes. This compares to the manufacturers quoted Vt repeatability of +/-20% with the Breas 30 ventilator. No attempt has been made to compare the systems directly and this was not an objective of the current study.

*Impact of shortening the Nafion line on measure SF6 signal*

Methods: 0.2% SF_6_ was bubbled through the smaller of the lung model tanks, heated to 57^o^C. Following thorough flushing, [SF_6_] was measured on the exhaust side of this by placing the gas sample needle in the exhaust port of the lung model. To measure dry gas, the sample needle was pierced through a plastic connector on the supply side of the model. Thus the same gas could be sampled dry from the tank or humidified after passage through warmed water. Measured humidity on the exhaust side was 70% at the point the gas was sampled. This was performed using the shortened (87cm) Nafion gas sample line, and repeated using the standard (170cm) line. Gas was sampled for at least 20 seconds of stable signal (2000 data points).

Results:

| **Condition** | **Nafion length (cm)** | **Humidity %** | **Mean [SF_6_] %** | **SD [SF_6_] %** |
| --- | --- | --- | --- | --- |
| Dry | 87 | 0 | 0.2073 | 0.0002 |
| Humidified | 87 | 70 | 0.2071 | 0.0003 |
| Humidified | 170 | 70 | 0.2072 | 0.0003 |

**Table S1:** Effect of humidifcation and length of Nafion gas sample line on measured SF_6_ signal.

There was negligible effect on [SF_6_] of adding humidication (Table S1). The difference between dry and humidified conditions, was 0.00024% absolute, or 0.1% of the baseline signal. The longer Nafion line resulted in an intermediate mean value.
